# Vertical variation of mixing within porous sediment beds below turbulent flows

**DOI:** 10.1002/2015WR018274

**Published:** 2016-05-07

**Authors:** I. D. Chandler, I. Guymer, J. M. Pearson, R. van Egmond

**Affiliations:** ^1^ HR WallingfordWallingfordUK; ^2^ School of EngineeringUniversity of WarwickCoventryUK; ^3^ Unilever Safety and Environmental Assurance CentreColworth Science ParkSharnbrookUK

**Keywords:** water‐sediment exchange, mixing

## Abstract

River ecosystems are influenced by contaminants in the water column, in the pore water and adsorbed to sediment particles. When exchange across the sediment‐water interface (hyporheic exchange) is included in modeling, the mixing coefficient is often assumed to be constant with depth below the interface. Novel fiber‐optic fluorometers have been developed and combined with a modified EROSIMESS system to quantify the vertical variation in mixing coefficient with depth below the sediment‐water interface. The study considered a range of particle diameters and bed shear velocities, with the permeability Péclet number, $Pe_{K}$ between 1000 and 77,000 and the shear Reynolds number, $Re_{*},$ between 5 and 600. Different parameterization of both an interface exchange coefficient and a spatially variable in‐sediment mixing coefficient are explored. The variation of in‐sediment mixing is described by an exponential function applicable over the full range of parameter combinations tested. The empirical relationship enables estimates of the depth to which concentrations of pollutants will penetrate into the bed sediment, allowing the region where exchange will occur faster than molecular diffusion to be determined.

## Key Point

- Sediment‐water exchange, laboratory study, mixing coefficients

## 1 Introduction

The impact of chemical pollutants on the environment, particularly aquatic ecosystems, has been the focus of much research in recent years. River ecosystems include the macro‐invertebrate benthic communities which may be strongly influenced by contaminant concentrations, both in the pore water and adsorbed to fine sediment particles [*Bottacin‐Busolin et al*., 2009]. Different modeling approaches have been proposed including transient storage, *Runkel* [1998] and risk assessment models based on the “impact zone” concept [*McAvoy et al*., 2003]. A knowledge of the movement of soluble chemical pollutants from the water column across the sediment‐water interface, and then into the sediment bed, or vice versa, may be required. This process of mass transfer across the sediment‐water interface is referred to as hyporheic exchange. *Boano et al*. [2014] provide a comprehensive overview of the hyporheic zone as one of the key elements of river corridors where water exchange is characterized by a wide range of spatial and temporal scales. The review discusses the transport of water, heat, dissolved and suspended compounds. Models, currently employed [*Technical Guidance Document (TGD)*, 2003] to predict exposure in sediments, are based on an assumption of equilibrium partitioning between dissolved and suspended‐particle‐sorbed phase in the water column. The bed sediment is assumed to consist of deposited suspended solids (with associated sorbed chemicals). Direct solute interactions with the bed, via diffusive or advective transfer from the water column to sediment pore water, are not taken into account. When an exchange coefficient has been included in modeling [*Fries*, 2007], it has been assumed to be constant with depth below the sediment‐water interface.

Numerous studies [*Marion et al*., 2002; *Packman et al*., 2004; *Tonina and Buffington*, 2007; *Rehg et al*., 2005; *Ren and Packman*, 2004] have shown significant mass transfer across the sediment‐water interface into the hyporheic zone. These studies investigated the interface exchange and not the variation in mixing with depth. *Hester et al*. [2013] numerically investigated the mixing zone thickness, in particular the mixing‐defined hyporheic zone on river beds and conclude that dispersivity is a critical parameter for which data are needed for shallow sediments. *Nagaoka and Ohgaki* [1990] and *Shimizu et al*. [1990] both showed a reduction in mixing with depth below the sediment‐water interface, but the studies were limited to depths of a few particle diameters below the interface.

Previous laboratory studies have used recirculating flumes to investigate hyporheic exchange. These allow the effect of bed forms to be studied, however they generally require large volumes of sediment and an extensive setup period, which restrict the range of conditions that can be tested in one series. Smaller volumes of both sediment and water would significantly reduce the time required, however this is difficult to achieve in a laboratory flume whilst maintaining a realistic physical scale. This paper explores the potential of utilizing an experimental tool to examine some of these processes.

The EROSIMESS System (shortened to erosimeter) is an instrument designed to generate realistic scale boundary shear and turbulence to investigate critical bed shear stress of sediment beds [*Liem et al*., 1997; *Spork et al*., 1997]. The erosimeter was originally developed at The Institute of Hydraulic Engineering and Water Resources Management, Aachen University of Technology in Germany (IWW, RWTH) and has been modified previously to study the effect of sediment resuspension on dissolved oxygen content of river water [*Jubb et al*., 2001] and to quantify hyporheic exchange coefficients [*Chandler et al*., 2012]. This paper describes further developments, to include both in‐flow and in‐bed fluorometric measurements. The erosimeter was then employed to record temporal concentration variations, both above and within‐bed, from an initial concentration of interstitial bed fluid, subject to a range of applied shear stresses. New results quantifying both an interface exchange coefficient and the vertical variation in mixing within porous sediment beds below the sediment‐water interface are presented and empirical scaling relationships explored.

## 2 Previous Work

Several different approaches have been taken to predict and/or model the interface exchange between flowing water and a porous sediment bed, the hyporheic exchange. Conceptual physical models have been studied, such as: a pumping model [*Elliott and Brooks*, 1997; *Tonina and Buffington*, 2007], slip flow model [*Fries*, 2007], a transient storage [*Hart*, 1995; *Runkel*, 1998; *Johansson et al*., 2001; *Wörman*, 2000; *Jonsson et al*., 2003; *Marion et al*., 2003] and a 1‐D vertical diffusion model [*Wörman et al*., 2002; *Packman et al*., 2004; *Habel et al*., 2002]. Additionally empirical scaling relationships [*Richardson and Parr*, 1988; *Packman and Salehin*, 2003; *O*'*Connor and Harvey*, 2008] have provided useful insights into the relative magnitude of contributing parameters. This approach will be taken here to investigate the spatial variation of in‐sediment mixing. The following sections describe the basic properties and parameters previously employed in estimating hyporheic exchange, how these parameters have been combined within empirical relationships to estimate the sediment‐water exchange coefficient and finally, how in‐bed mixing processes, driven by turbulence generated at the sediment‐water interface, have been quantified using a Fickian analogy.

### 2.1 Fundamental Parameters

Previous studies investigating hyporheic exchange have shown that permeability ( K), bed shear velocity ( $u_{*}$) and roughness height ( $k_{s}$) are important parameters affecting hyporheic exchange. There are several formulae available to predict permeability. The Kozeny‐*Carman* equation [*Carman*, 1937], cited [*Freeze and Cherry*, 1979] gives

(1) $$K_{c}=\left(\frac{\rho_{w}g}{\mu }\right)\left(\frac{\theta^{3}}{{\left(1-\theta \right)}^{2}}\right)\left(\frac{d_{g}^{2}}{180}\right)$$

where $K_{c}$ is the hydraulic conductivity, $\rho_{w}$ is the density of the fluid, g is gravitational acceleration, μ is dynamic viscosity, θ is porosity and $d_{g}$ is the mean grain diameter.

Hydraulic conductivity can be converted to permeability (2), which when combined with (1), results in (3) that can predict the permeability of a sediment [*O'Connor and Harvey*, 2008].

(2) $$K=\frac{K_{c}\nu }{g}$$

where ν is the kinematic viscosity ( $\nu =\mu /\rho_{w}$).

(3) $$K\;=\;5.6\times 10^{-3}\frac{\theta^{3}}{{\left(1-\theta \right)}^{2}}d_{g}^{2}$$

Equation (1) is derived from Darcy's law and the packing of spheres, with the addition of an experimentally derived constant [*Bear*, 1972]. *Carman* [1937] states that (1) is valid for nonspherical particles in the streamline (laminar) flow region with an error of 10–20%. Equation (3) is used by *O'Connor and Harvey* [2008] to derive their scaling relationship when the permeability was not stated in previous studies. It has been used in this study to validate the in situ permeability measurements. To provide an assessment of potential subseafloor pore water advection, *Wilson et al*. [2008], investigated the potential for employing grain size as a predictor of permeability in coastal marine sand and recommend permeability‐grain size relationships may be useful, but that a larger database is required.

The bed shear velocity, $u_{*}$ is defined [*Fischer et al*., 1979; *Tennekes and Lumley*, 1972] as

(4) $$u_{*}=\sqrt{\frac{\tau }{\rho_{w}}}$$

where τ is the bed shear stress, which combined with (5), derived from the rate of interchange of momentum in the Reynolds stress model of turbulence [*Tennekes and Lumley*, 1972], gives

(5) $$\partial F=\rho_{w}\bar{u^{\prime }v^{\prime }}\partial A$$

(6) $$u_{*}=\sqrt{\bar{u^{\prime }v^{\prime }}}$$

where $u^{\prime }$ and $v^{\prime }$ are the instantaneous velocity fluctuations in the horizontal and vertical directions respectively. *Tennekes and Lumley* [1972] state that if viscous effects are negligible, the velocity fluctuations are correlated and the average vertical flow at the sediment‐water interface is zero, then (6) is valid at any vertical position within the flow.

The roughness height, $k_{s}$ is a function of both the sediment grain diameter and any bed‐forms present. It allows comparison, through a single parameter, of experiments that have flat beds with those where bed‐forms were used. *van Rijn* [1984] defined the roughness height as

(7) $$k_{s}=3d_{90}+1.1\Delta \left(1-e^{-25\Delta /\lambda }\right)$$

where $d_{90}$ is the particle size such that 90% of the particles are finer, Δ is the bed‐form amplitude and λ is the bed‐form wavelength. For flat‐bed experiments,  Δ=0 and (7) reduces to

(8) $$k_{s}=3d_{90}$$

### 2.2 Interface Exchange Studies

Several studies have been conducted to derive empirical scaling relationships to describe the solute exchange at the sediment‐water interface. All studies use the same general methodology, based on Fick's second law in one dimension

(9) $$\frac{\partial C}{\partial t}=D\frac{\partial^{2}C}{\partial y^{2}}$$

where C is the solute concentration, t is time, D mixing coefficient and y is the vertical coordinate.

*Richardson and Parr* [1988] conducted flume experiments with glass beads for three flow depths, four velocities and five bead diameters, representing fine to very coarse sands. For an initially saturated bed, they measured the tracer concentration at the effluent weir throughout the 30 min experiments. From these temporal concentration measurements, after an initial non‐Fickian phase, they showed that

(10) $$\frac{D_{e}}{{D^{\prime }}_{m}}=6.59\times 10^{-5}{Pe}_{K}^{2}$$

where $D_{e}$ is the effective interface mixing coefficient, $D_{m}^{\prime }$ is the molecular diffusion coefficient through the sediment pore water and $Pe_{K}$ is the permeability Péclet number

(11) $$Pe_{K}=\frac{u_{*}\sqrt{K}}{D_{m}^{\prime }}$$

*Packman and Salehin* [2003] used seven published data sets to derive a scaling relationship, covering a much wider range of sediment and flow conditions than those used by *Richardson and Parr* [1988]. They proposed two scaling relationships, the first combining the permeability, the dynamic pressure head and the sediment porosity was found to be appropriate for larger diameter material and fitted data over more than three orders of magnitude. It did not however hold for fine sands and so they reported an alternative scaling relationship, which holds for almost five orders of magnitude

(12) $$D_{e}={\left(Re.d_{g}\right)}^{2}$$

where Re is the stream Reynolds number $Re=\frac{Uh}{\nu }$ where U is the average velocity in the main stream and h is the flow depth.

A practical method for predicting exchange coefficients across the sediment‐water interface over a wide range of sediment and flow conditions is the scaling relationship proposed by *O'Connor and Harvey* [2008]. It is derived from 11 previously published data sets covering numerous different sediment characteristics, flow parameters and topographies. *O'Connor and Harvey* [2008] proposed

(13) $$\frac{D_{e}}{{D^{\prime }}_{m}}=\left\{\begin{array}{ll} {5\times 10^{-4}Re}_{*}{Pe}_{K}^{6/5} & \text{for}\;{Re}_{*}{Pe}_{K}^{6/5}\geq 2000\\ 1 & \text{for}\;{Re}_{*}{Pe}_{K}^{6/5}<2000 \end{array}\right.$$

where $Re_{*}$ is the shear Reynolds number, $Re_{*}=\frac{u_{*}k_{s}}{\nu }$ and the inverse of the scaling constant $\left(5\;x\;10^{-4}\right)$ provided a threshold value in transport conditions ( $Re_{*}Pe_{K}^{6/5}$ = 2000), below which transport was governed by molecular diffusion, resulting in $D_{e}/D_{m}^{\prime }$ = 1. A limitation of this approach, when investigating fundamental relationships, is that both the nondimensional numbers, the shear Reynolds number, $Re_{*}$ and the permeability Péclet number, $Pe_{K}$ consist of independent variables, as both are functions of the bed shear velocity, $u_{*}$.

The data collated by *O'Connor and Harvey* [2008] resulted from different experimental setups. All the studies used recirculating flumes, but the initial location of the solute tracer and the sampling location (either in‐bed or water column) was different. This resulted in different equations being used to analyze the data. For a temporal concentration profile obtained from an instrument positioned within the water column and tracer initially located in the sediment pore water (in‐bed), *O'Connor and Harvey* [2008] calculated the exchange coefficient across the sediment‐water interface from

(14) $$D_{e}={\left(\frac{\sqrt{\pi }}{2C_{0,s}}\frac{dM_{w}}{d\left(t^{1/2}\right)}\right)}^{2}$$

where $C_{0,s}$ is the initial solute concentration within the sediment pore water, $dM_{w}/d(t^{1/2})$ is the “initial slope” taken from the temporal concentration profile, where $M_{w}$ is the accumulated mass of solute tracer in the water column and $t^{1/2}$ is the square root of time. However, *O'Connor and Harvey* [2008] do not specify what portion of the profile corresponds to the “initial slope.” Similar approaches have been adopted by *Packman et al*. [2004], *Chandler et al*. [2012], and others.

### 2.3 In‐Bed Studies

A few studies have measured temporal concentration profiles within the bed sediment. *Liu et al*. [2014] provide a review of recent advances in the measurement of the diffusive flux of chemicals at the sediment‐water interface, describing a new sampler [*Liu et al*., 2013] which, unlike a conventional benthic chamber, does not need to assume a linear concentration gradient, though an estimation of the chemical diffusion coefficient in the overlying water is still required. *Cho et al*. [2010] employed temperature as a tracer and studied the advective pore water movement in the top 0.60 m sediment layer in marine mudflat sediments. In the limiting case, with no net advection, they report the best‐fit depth‐averaged mechanical dispersion coefficient was 2.2.10^−7^ m^2^/s, with a range between 0.9 and 5.6.10^−7^ m^2^/s.

*Nagaoka and Ohgaki* [1990] and *Shimizu et al*. [1990] showed a reduction in mixing coefficient with depth, over a few particle diameters below the interface. They employed large diameter glass spheres (geometric mean particle diameter, $d_{g}$ ≥ 17 mm) for the sediment bed. Neither study quantified the variation in mixing coefficient with depth, as the primary focus of both papers was on understanding the flow through porous media. *Nagaoka and Ohgaki* [1990] and *Shimizu et al*. [1990] used different analysis techniques to obtain mixing coefficients from the in‐bed concentration profiles. *Shimizu et al*. [1990] use the time at which the measured concentration equals 1/ e of the equilibrium mixing concentration (where e is the base of the natural logarithm) to fit an analytical solution of Fick's second law (13), thus obtaining a mixing coefficient from one in‐bed profile. This technique is susceptible to experimental noise, as only one point is used and it does not account for variations in the mixing coefficient depth.

*Nagaoka and Ohgaki* [1990] also used an analytical solution to Fick's second Law (9). They solved this using initial and boundary conditions:

(15) C(0,y) = 0

(16) $$C\left(t,0\right)=\;f\left(t\right)$$

(17) $$C\left(t,-L^{-}\right)=\;C\left(t,-L^{+}\right)$$

(18) $$\underset{y\to -\infty }{\lim}C(t,y)=0$$

(19) $$D\;=\;D_{1}\left(0<y<-L\right)$$

(20) $$D\;=\;D_{2}\left(-L<y<-\infty \right)$$

(21) $$D_{1}{|\frac{\partial C}{\partial y}}_{y=-L-}=D_{2}{|\frac{\partial C}{\partial y}}_{y=-L+}$$

where L is the vertical distance between sensors. This corresponds to the scenario where two different layers, with different mixing coefficients ( $D_{1}$ in the upper and $D_{2}$ in the lower) are acting at y=−L. The upper layer is from y=0 to y=−L and the lower layer is from y=−L to y=−∞. Substituting y=−L into the analytical solution to Fick's second law, *Nagaoka and Ohgaki* [1990] obtained the concentration change at the interface between the two layers, *C_A_* as

(22) $$C_{A}\left[f\left(t\right),\;D_{1},\;D_{2},L\right]=\frac{L}{\left(b+1\right)\sqrt{\pi D_{1}}}\times \int_{0}^{t}\frac{f\left(\varepsilon \right)}{{\left(t-\varepsilon \right)}^{\frac{3}{2}}}\sum_{j=0}^{N}c^{j}\left(2j+1\right)\times \exp\left(-\frac{{\left(2j+1\right)}^{2}L^{2}}{4D_{1}\left(t-\varepsilon \right)}\right)d\varepsilon$$

where $b=\sqrt{\frac{D_{2}}{D_{1}}}$ and $c=\frac{b-1}{b+1}$

Equation (22) expresses how the concentration at the interface between the two layers changes when the concentration at the upper edge of the upper layer, f(t), changes. This equation can be applied if the change in concentration at the top of the upper layer and both mixing coefficients are known. To analyze experimental data, the calculated profile can be optimized to give the best fit to the measured data by varying $D_{1}$. $D_{2}$ can be fixed from analysis of the region below that currently being studied.

Applying this to several layers creates a challenge with analyzing the lowest region, because $D_{2}$ is not known and cannot be obtained from the analysis of a lower region. However taking the limit $D_{1}\to D_{2}$, *Nagaoka and Ohgaki* [1990] defined another function

(23) $$C_{B}\left[f\left(t\right),D_{1}\right]=\frac{L}{2\sqrt{\pi D_{1}}}\int_{0}^{t}\frac{f(\varepsilon )}{{\left(t-\varepsilon \right)}^{3/2}}\times \exp\left(-\frac{L^{2}}{4D_{1}(t-\varepsilon )}\right)d\varepsilon$$

Here the assumption is that the mixing coefficient for the upper layer is the same as that in the lower layer. The analysis methodology therefore starts by optimizing $D_{1}$ in (23) so that the closest match is found between the predicted profile and measured concentration profile from the lowest instrument position. Once $D_{1}$ is obtained between the lowest pair of instruments, it can be used as $D_{2}$ in (22), and $D_{1}$ can be optimized between the next lowest instrument pair.

## 3 Experimental Setup

**Figure 1:**
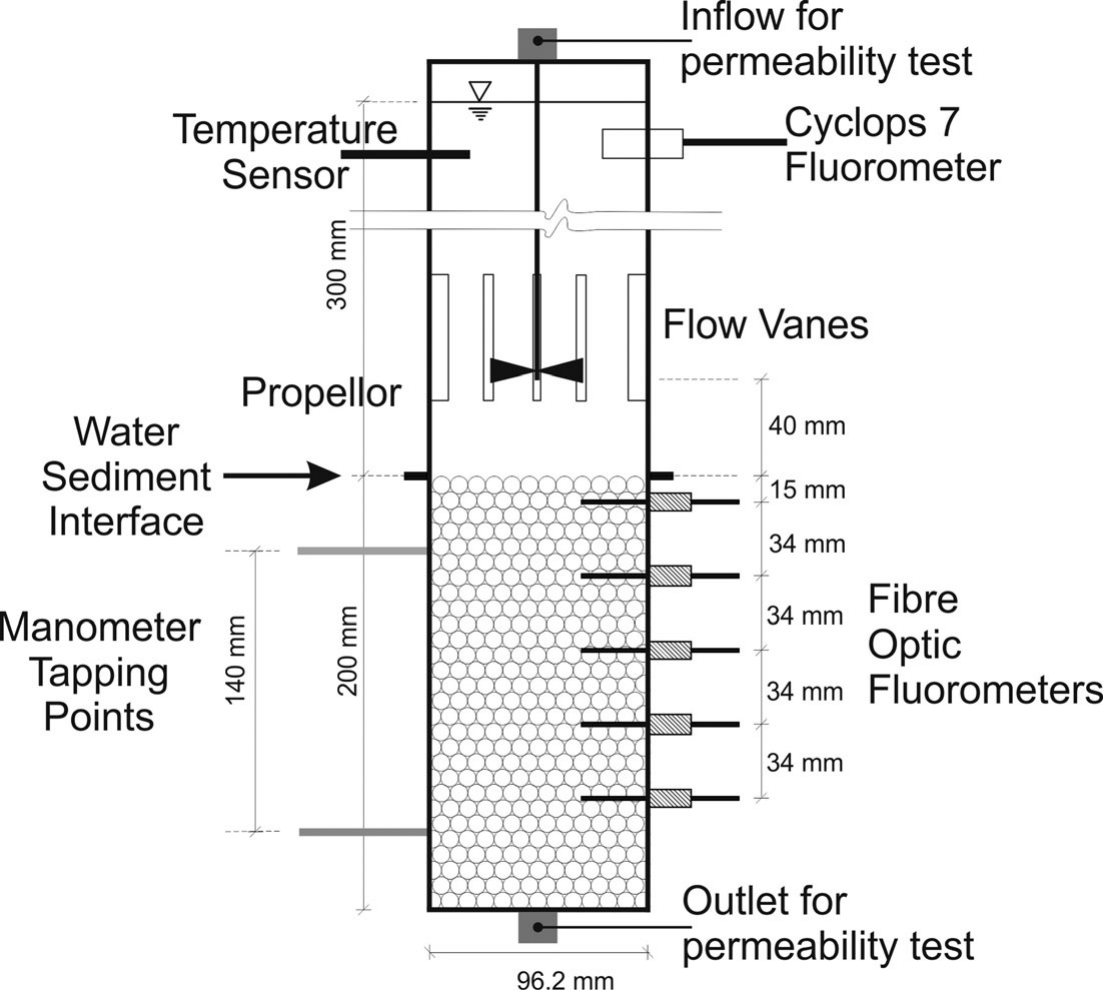
Schematic of Erosimeter Experimental Set‐Up.

An erosimeter was modified to improve the placement of sediment, provide side access for instrumentation in the base section and to incorporate an in situ permeability test. Figure 1 shows the redesigned erosimeter, with a flanged connection between the main section and base at the sediment‐water interface, and outlet in the base for the permeability testing.

The main section is 300 mm high with an internal diameter of 96.2 mm. A Turner Designs Cyclops 7 fluorometer and a temperature sensor were positioned on opposite sides 60 mm below the top. The 200 mm tall base section had the same diameter as the main section. Fiber ‐optic fluorometers were aligned vertically at −0.015, −0.049, −0.083, −0.117, and −0.151 m below the top of the base section, the sediment‐water interface.

A motor sits on top of the main section with a 260 mm long shaft bringing the 20 mm diameter tri‐bladed propeller to 40 mm above the sediment‐water interface. Six baffles around the circumference, at the height of the propeller, create a uniform bed shear stress at the sediment surface [*Liem et al*., 1997].

The propeller speed is calibrated to the bed shear velocity ( $u_{*}$) through observing the onset of sediment motion, where “the grains roll over the sediment surface, being moved a significant distance” for single size sediments. Thereby obtaining the critical bed shear stress which is used to estimate bed shear velocity through the *van Rijn* [1984] criteria, as used by *Jubb et al*. [2001].

### 3.1 Experimental Procedure

Each test consisted of five main stages. The first stage was to place a homogeneous concentration of Rhodamine throughout the bed into the base section and take a calibration reading for the fiber optic fluorometers. Next, the main section was placed and filled with clean de‐aired water. The motor was then installed, switched on and the tracer experiments allowed to run. Once the tracer experiment was complete the motor was stopped and replaced by the constant head permeability test apparatus. The permeability test was then conducted on the in situ bed sediment.

The test series consisted of five different bed shear velocities and five different sediment diameters in various combinations which are given in Table 1, along with the number of tests conducted for each parameter combination. Some combinations could not be tested without causing sediment motion, which was undesirable in this study, and are indicated with a “‐ ”in Table 1. The sediment consisted of single size solid soda glass spheres with a quoted density of 2530 kg/m^3^. The range of particle diameters, including the mean particle diameters, is given in Table 2. The solute tracer used was Rhodamine WT, a fluorescent tracer developed in the 1960s (US patent 3, 367.946) and was initially placed in the interstitial fluid, with clean water in the water column.

**Table 1:** Test Combinations

Propeller Speed (rpm)	Number of Tests					Bed Shear Velocity $u_{*}$ (m/s)	
	Mean Particle Diameter, *d_g_*(mm)						
	5.000	1.850	0.625	0.350	0.150	Calibrated	PIV (19)
440	3	‐	‐	‐	‐	0.0406	0.0427
329	2	2	‐	‐	‐	0.0296	0.0266
226	2	2	‐	‐	‐	0.0194	0.0166
179	2	2	2	1	‐	0.0147	0.0147
124	2	2	2	1	1	0.0093	0.0091

**Table 2:** Sediment Properties

Mean Particle Diameter, *d_g_* (mm)	90% Larger, *d_10_* (mm)	90% Smaller, *d_90_* (mm)	Permeability, K (10^−10^ m^2^)	
			Measured	Calculated (3)
5.000	4.700	5.300	223	107
1.850	1.700	2.000	30.6	20.4
0.625	0.500	0.750	3.12	3.18
0.350	0.300	0.400	0.98	1.38
0.150	0.100	0.200	0.18	0.46

### 3.2 Particle Image Velocimetry

To confirm the flow field within the erosimeter, particle image velocimetry (PIV) measurements were undertaken. The aims were to qualitatively asses the flow field within the system, to establish the uniformity of the flow field at the sediment‐water interface and to relate the velocity field to the bed shear velocity ( $u_{*}$) obtained during the sediment motion calibration. Two experimental setups were employed: the first with a vertical light sheet (VLS) and the second with a horizontal light sheet (HLS). Further information on the PIV setup can be found in *Chandler* [2012].

Five different propeller speeds were investigated, which correspond to the bed shear velocities employed in the dye tracing experiments. Horizontal light sheets at 3 mm, 13 mm and 23 mm above the fixed bed were used, along with one vertical light sheet position, across the centre of the erosimeter between the bed and the propeller, for both fixed and mobile beds.

Raw images were processed using DaVis 7.2 (a LaVision product) to produce velocity vector fields. From these, temporal average vector fields were generated and the instantaneous velocity fluctuations calculated. The velocity components for each point within the vector fields were averaged using

(24) $$\bar{u}=\frac{1}{N}\overset{N}{\underset{i=1}{\sum }}u_{i}$$

where $\bar{u}$ is the temporally averaged velocity in the x‐direction and N is the number of vector fields. The vector fields already account for the time step between images, so time is not explicitly used in the averaging, only the number of vector fields. The same equation was also used for the vertical velocities ( v) and the other horizontal component ( w) in the z‐direction.

The vertical light sheet data contain components w and v, whilst the horizontal light sheet data contain components u and w. As discussed in 2.1, the bed shear velocity can be calculated from the velocity fluctuations. In the coordinate system imposed here, and given the flow field discussed below, (6) becomes

(25) $$u_{*}=\sqrt{\bar{w^{\prime }v^{\prime }}}$$

This requires simultaneous velocity components from both the horizontal and vertical light sheets, at the line across the erosimeter where the sheets would intersect. However, as the measurements were not conducted simultaneously, the instantaneous fluctuations in the z and y directions are not concurrent.

**Figure 2:**
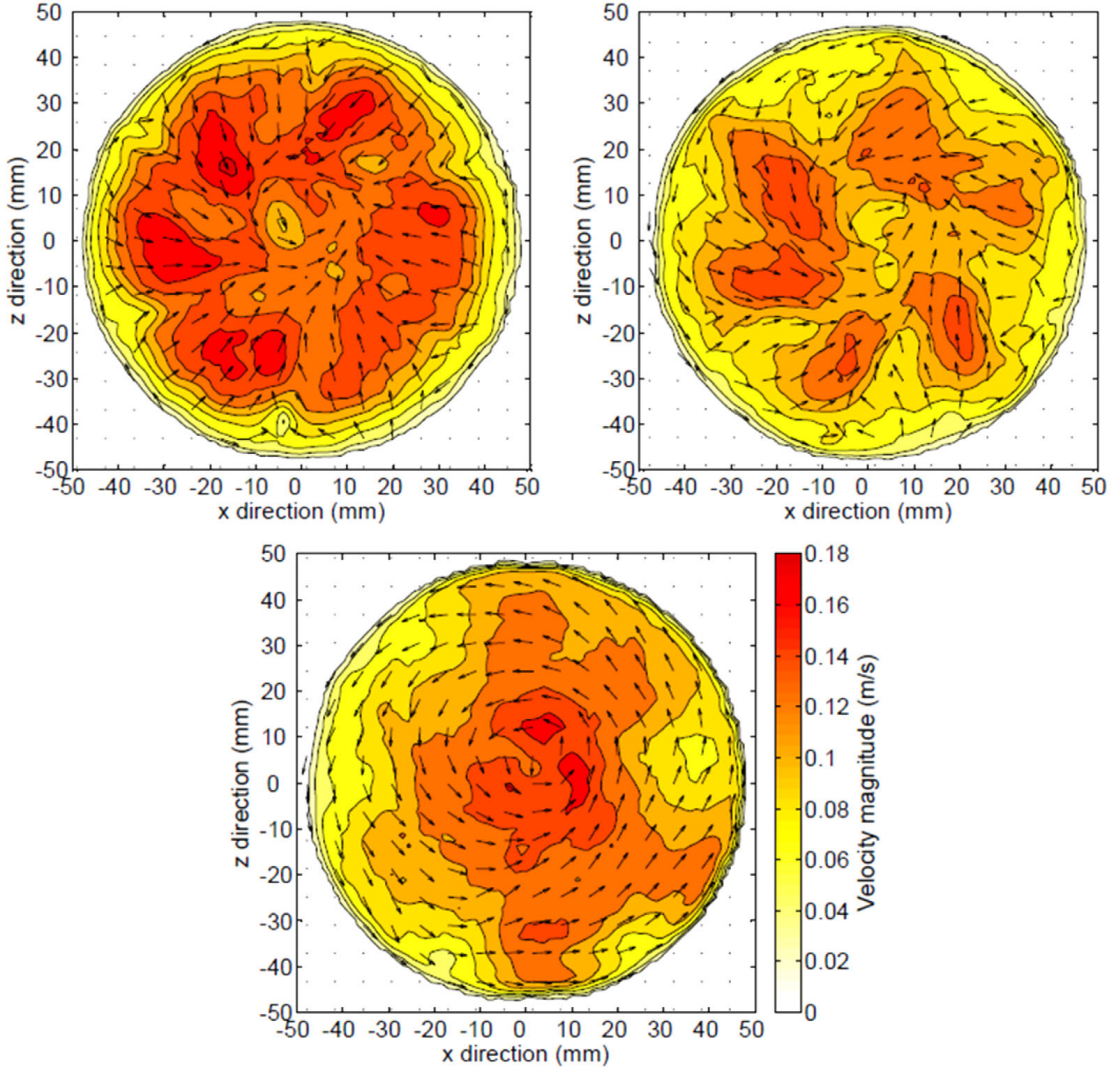
Time averaged horizontal light sheet vector fields at different heights above the bed.

Bed shear velocity is often used as a measure of turbulent intensity and is taken as indicative of turbulence in all directions if turbulence is assumed to be homogeneous. Given the flow field above the bed in the erosimeter shown in Figure 2, the wall shear around the edge of the erosimeter should be similar to the bed shear, assuming that the turbulence is homogeneous. Therefore the assumption has been made that the bed shear velocity can be calculated from the velocity fluctuations in the x and z directions using the horizontal light sheet data from 3 mm above the bed. Therefore (25) becomes

(26) $$u_{*}=\sqrt{\bar{u^{\prime }w^{\prime }}}$$

**Figure 3:**
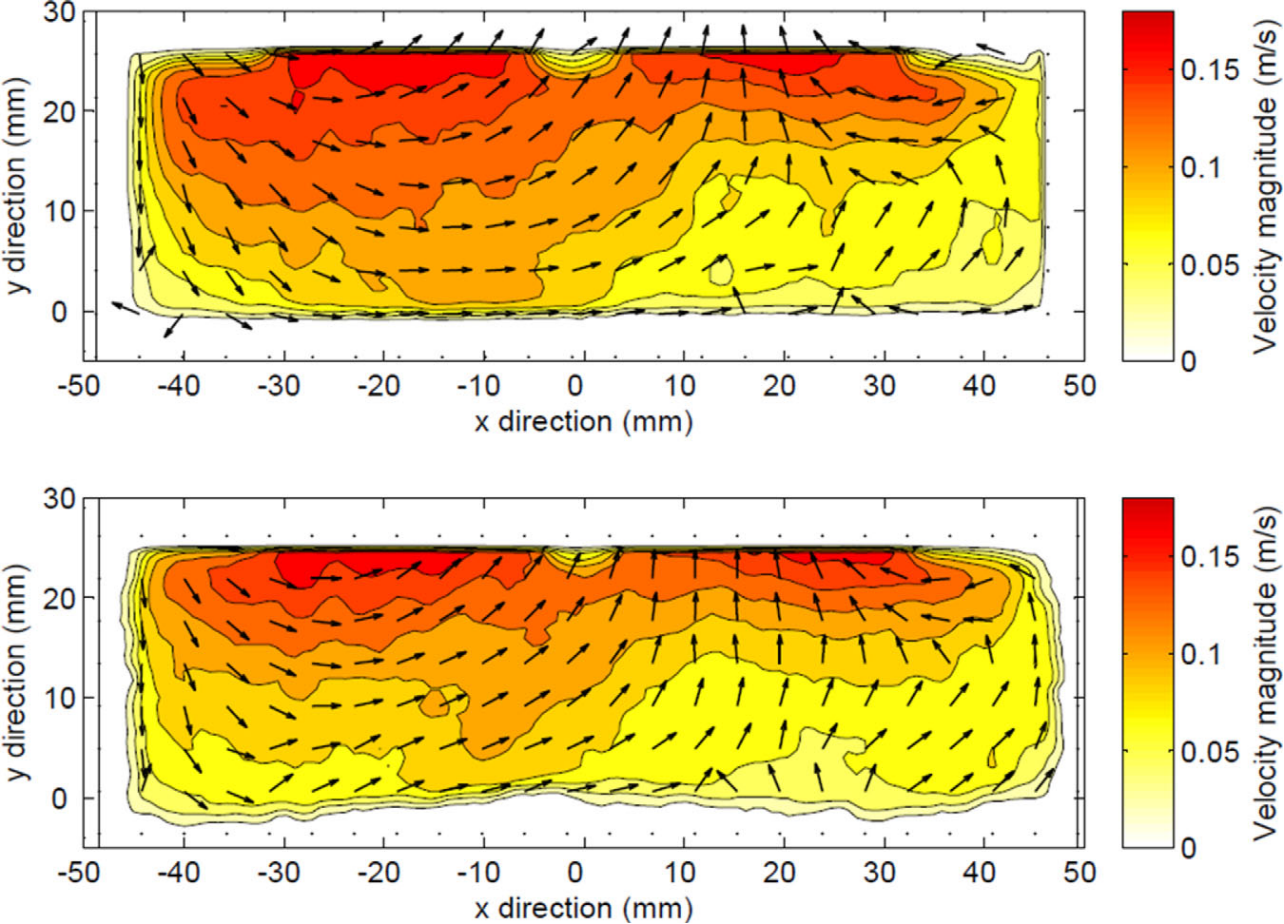
Time averaged vertical light sheet vector fields over fixed and mobile bed.

### 3.3 Fluorometry

In‐bed concentration measurements were taken using fiber‐optic fluorometers. The fiber‐optic fluorometers had a head diameter of 4 mm. A mesh cover (30 mm long by 4 mm) was positioned over the end of the fiber to create a measurement volume of approximately 0.23 ml. The excitation source was a green laser diode and the emissions detector was a photo multiplier tube (PMT) with appropriate optical filters for Rhodamine WT. The signal from the PMT was passed through a low pass filter, with a cut off frequency of 30 Hz, to reduce noise from the mains power supply, whilst still capturing the expected rate of concentration change.

The fiber optic fluorometers were calibrated in situ for each test using a two point calibration, whilst the Cyclops 7 fluorometer, used for water column concentration measurements, was calibrated before and after the test series. All the fluorometers had an accuracy of 1 ppb or better.

### 3.4 Permeability Test

The base section of the erosimeter includes a drain so that a constant head permeability test can be conducted [*British Standard*, 1990, 1377‐5], in situ, after solute trace experiments had been undertaken. A cap, connected to the constant head source, is placed on top of the main section, replacing the motor and housing. Manometer gland points, 140 mm apart in the base, are used to measure the hydraulic gradient ( I) within the sediment bed. This gradient is used to calculate hydraulic conductivity of the sediment using

(27) $$K_{C}=\left(\frac{Q}{I}\right)\left(\frac{R_{T}}{A_{s}}\right)$$

where Q is flow rate (ml/s), I is hydraulic gradient ( h/y) with h the difference in manometer level (mm) and y the distance between manometer gland points (mm), $R_{T}$ is the temperature correction factor obtained from *British Standard* [1990] 1377‐5 and $A_{s}$ is the cross‐sectional area of the sample (mm^2^). The hydraulic conductivity is converted into a permeability using (2).

The permeability measurements taken after the tracer experiments show good agreement with the calculated permeability using (3), shown in Table 2. The higher than expected permeability for large diameter spheres could be due to nonlaminar flow conditions within the permeability tests. This would invalidate the assumption of Darcy flow in the derivation of (3) and lead to the discrepancy. The difference between the measured and calculated permeability for the small diameter sediment is within the 10–20% bound suggested by *Carman* [1937].

## 4 Results

### 4.1 PIV Experiments

The flow field within the erosimeter is complex and changes with height above the bed. However, the spatial velocity distribution is independent of propeller speed, which only changes the magnitude of the velocities. The field is relatively uniform at the bed with slightly higher velocities in the centre and lower velocities around the outside near the wall. The velocities obtained from the PIV data are comparable to those reported by *Liem et al*. [1997].

Example time averaged horizontal light sheet vector fields at three heights above a fixed bed for propeller speed 440 rpm are shown in Figure 2. Raw images were recorded at 1000 fps for 3.2 s for each propeller speed. Close to the bed, 3 mm above in Figure 2c, the flow is rotational, without any inward motion that is seen at 23 mm above the bed, Figure 2a. There are higher velocities in the centre and lower ones around the edge at the bed. The mean velocity is 0.11 m/s, with most velocities within ± 40% of the mean. Figure 2 suggests an approximately uniform circulating flow field at the bed, which has similar velocities at the wall to those seen at the bed in vertical light sheet data shown in Figure 3. This suggests the assumption made in deriving equation (19) is valid and the velocity fluctuations from the horizontal light sheet 3 mm above the bed can be used to estimate the bed shear velocity. Table 1 shows that there is close agreement between the bed shear velocity calculated from the PIV data and those calculated independently from the sediment motion calibration.

### 4.2 Trace Experiments

**Figure 4:**
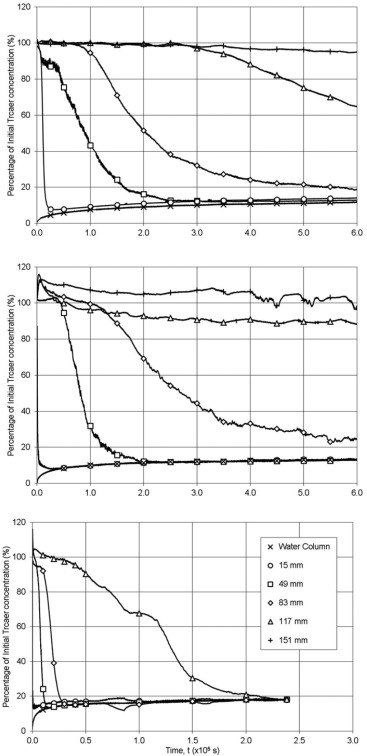
Examples of raw data.

Examples of calibrated temporal concentration profiles from the Cyclops 7 fluorometer within the main body, the water column, and the fiber optic fluorimeters located within the sediment bed are provided in Figure 4. The increase in concentration within the water column is evident, showing that not every experiment was run until full equilibrium conditions were developed. The effect of spatial difference in instrument position below the sediment‐water interface is clearly visible, with the instruments closer to the interface showing more rapid reductions in concentration, and hence mixing, than those further away. Comparing Figures 4a and 4b illustrates the increase in exchange from the initial high concentration within the bed to the water column resulting from increased particle diameter, for the same bed shear velocity. In Figure 4a, for mean particle diameter of 0.00035 m, with a bed shear velocity of 0.015 m/s, it takes over an order of magnitude longer for the concentration 0.083 m below the sediment‐water interface to reach equilibrium compared with Figure 4c, for mean particle diameter of 0.00185 m, with a bed shear velocity of 0.0120 m/s.

The results in Figure 4c show that the exchange of solute tracer from the pore water starts to occur −0.015 m below the sediment‐water interface within 60 s of the test starting, whereas there is no significant reduction of concentration at −0.117 m until 11 h into the test. The noise on the dye traces, Figure 4b, in the profiles at −0.151 m is caused by slight temperature fluctuations, as an increase in temperature will cause a decrease in fluorescence [*Smart and Laidlaw*, 1977]. Although the temperature was recorded in the upper part of the water column during the experiments, no temperature correction has been applied as the noise is recorded only on the lower fluorometers and did not affect the analysis. The temperature throughout the tests was 21 ± 1°C.

## 5 Analysis

To assess and refine the analysis techniques applied to the new experimental data, a one‐dimensional implicit finite difference solution to Fick's second law of diffusion [*Crank and Nicolson*, 1947] was employed. Firstly, the model was used to investigate the proportion of the temporal concentration profile that should be included when calculating the initial slope used in the *O'Connor and Harvey* [2008] method for water column data. The model was then employed to check the robustness of the *Nagaoka and Ohgaki* [1990] method for analyzing in‐bed data and to identify the best goodness of fit parameter for optimizing the in‐bed mixing coefficient.

The proportion of the temporal profile that should be included when calculating the initial slope is not stated by *O'Connor and Harvey* [2008]. A sensitivity analysis was conducted using three different model simulations: a constant mixing coefficient; a distribution with seven discrete coefficients and an exponential spatially varying coefficient. Different percentages of the equilibrium, fully‐mixed concentrations were used to define the end of, or last value to be included in, the initial slope. The coefficients obtained from taking different percentages of the equilibrium concentration were compared with the coefficients specified in the model simulations. The $R^{2}$ values of the linear best fit lines used to obtain the gradient of the initial slope were also studied. This analysis indicated that using a value of 15% of the equilibrium concentration to define the end of the initial slope is the most consistent and accurate method. Further details are provided in *Chandler* [2012]. The sensitivity analysis was extended to three different experimental profiles. The highest $R^{2}$ values of the linear best fit to the experimental data correspond to between 20 and 30% of the equilibrium concentration. This analysis, combined with the analysis of one‐dimensional diffusion model simulations, concluded that a value of 25% of the equilibrium concentration should be used to define the initial slope in the analysis of the experimental data.

The *Nagaoka and Ohgaki* [1990] methodology, for analysis of the in‐bed data, has been evaluated using a seven discrete mixing coefficient zone model simulation. Temporal concentration profiles taken from spatial points that correspond to the change in mixing coefficient between the different zones were analyzed [*Chandler*, 2012]. Table 3 gives the output from the *Nagaoka and Ohgaki* [1990] analysis for the model data with and without random noise added. The noise was added to check the robustness of the method. The coefficient of determination, $R_{t}^{2}$, [Young et al., 1980] was used as the goodness of fit parameter between the measured and predicted profiles for the results presented in Table 3. This study showed that the application of the technique was not sensitive to noise and generally produced values to within 10% lower than the specified values.

**Table 3:** *Nagaoka and Ohgaki* [1990] Analysis of a Variable Coefficient Model

		In‐Bed Mixing Coefficient, *D* (10^−7^ m^2^/s)		
Profile Boundary, *y* (m)			From Analysis	
Upper	Lower	Specified	No Noise	With Noise
−0.025	−0.050	20.00	18.90	18.80
−0.050	−0.075	6.00	5.36	5.33
−0.075	−0.100	2.00	1.85	1.85
−0.100	−0.125	0.60	0.60	0.599
−0.125	−0.150	0.20	0.20	0.197

These analysis techniques were applied to the complete data set comprising temporal concentrations distributions recorded simultaneously in both the water column and at various depths within the sediment bed, as shown in Figure 1. Typical distributions are shown in Figure 4. The temporal concentration distribution recorded within the water column was analyzed using the data from the start of the experiment until a concentration of 25% of the equilibrium concentration was reached. Predicted in‐bed temporal distributions were fitted to the recorded data. Overall, for the 14 test cases considered, a total of 25 interface mixing coefficients were obtained from the in‐flow data and 78 in‐bed mixing coefficients were evaluated. These results are summarized in Table 4.

**Table 4:** Evaluated Mixing Coefficients

Test Number	Conditions			Interface Mixing Coefficient, *D_e_* (10^−7^ m^2^/s)	In‐Bed Mixing Coefficients, *D* (10^−7^ m^2^/s)			
	*d_g_* (m)	u_*_ (m/s)	*K* (10^−10^ m^2^)		−0.032 m	−0.066 m	−0.100 m	−0.134 m
1	0.005	0.0407	112.27	19.220		48.511	14.596	1.160
		0.0406	97.02	18.879		136.481	40.828	34.911
		0.0403	115.89	16.546		78.124	14.175	5.237
2		0.0298	106.91	12.944		37.926	7.183	1.728
		0.0304	102.49	14.462		86.667	5.876	1.717
3		0.0198	103.12	6.190		32.099	9.037	‐
		0.0200	102.34	7.801	120.389	13.172	1.212	0.121
4		0.0152	102.51	3.796	19.090	5.304	0.439	0.029
		0.0154	107.74	7.193	27.877	4.855	0.698	0.067
5		0.0101	109.05	4.133	16.228	4.327	0.142	0.026
		0.0100	108.77	3.264	12.762	3.257	0.361	0.027
6	0.00185	0.0298	20.68	‐	‐	4.897	0.594	0.202
		0.0299	20.31	4.861	9.845	3.976	0.461	0.043
7		0.0197	21.13	2.061	3.435	1.058	0.119	0.031
		0.0197	20.56	1.865	3.854	1.213	0.102	0.023
8		0.0153	20.18	0.879	‐	1.341	0.077	0.016
		0.0153	19.59	0.879	1.797	0.508	0.062	0.013
9		0.0099	20.35	0.292	‐	0.101	0.012	
		0.0098	20.26	0.328	0.957	0.173	0.019	
10	0.000625	0.0152	3.15	0.124	0.210	0.057	0.008	
		0.0153	3.18	0.096	0.187	0.052	0.010	
11		0.0101	3.20	0.029	0.096	0.028	‐	
		0.0099	3.21	0.042	0.127	0.028	‐	
12	0.00035	0.0152	1.69	0.025	0.102	0.019	‐	
13		0.0100	1.07	0.011	0.074	0.012	‐	
14	0.00015	0.0098	0.46	0.001	0.000	‐	‐	

## 6 Discussion

The main focus for this study is to identify appropriate empirical scaling relationships for both the interface mixing coefficient and the spatial variation of the in‐bed mixing coefficients. The unique data collected from the erosimeter have been compared to proposed scaling relationships and a new multiple linear regression analysis performed, employing the experimental variables of mean particle diameter and bed shear stress.

### 6.1 Interface Mixing Coefficient

Interface mixing coefficients range over four orders of magnitude, from around 1.0 × 10^−10^ m^2^/s for 0.00015 m diameter particles exposed to a 0.01 m/s bed shear velocity, to 2.0 × 10^−6^ m^2^/s for the largest diameter particles tested, 0.005 m under a bed shear velocity of around 0.04 m/s. All values are provided in Table 4. Good repeatability between tests is evident. There are small variations in the permeability, due to slight differences in the packing of the glass spheres and the propeller speed, from which the bed shear velocity is inferred. To ease comparison with previous work, interface mixing coefficients have been nondimensionalized using the molecular diffusion coefficient in sediment pore water, ${D\prime }_{m}$.

**Figure 5:**
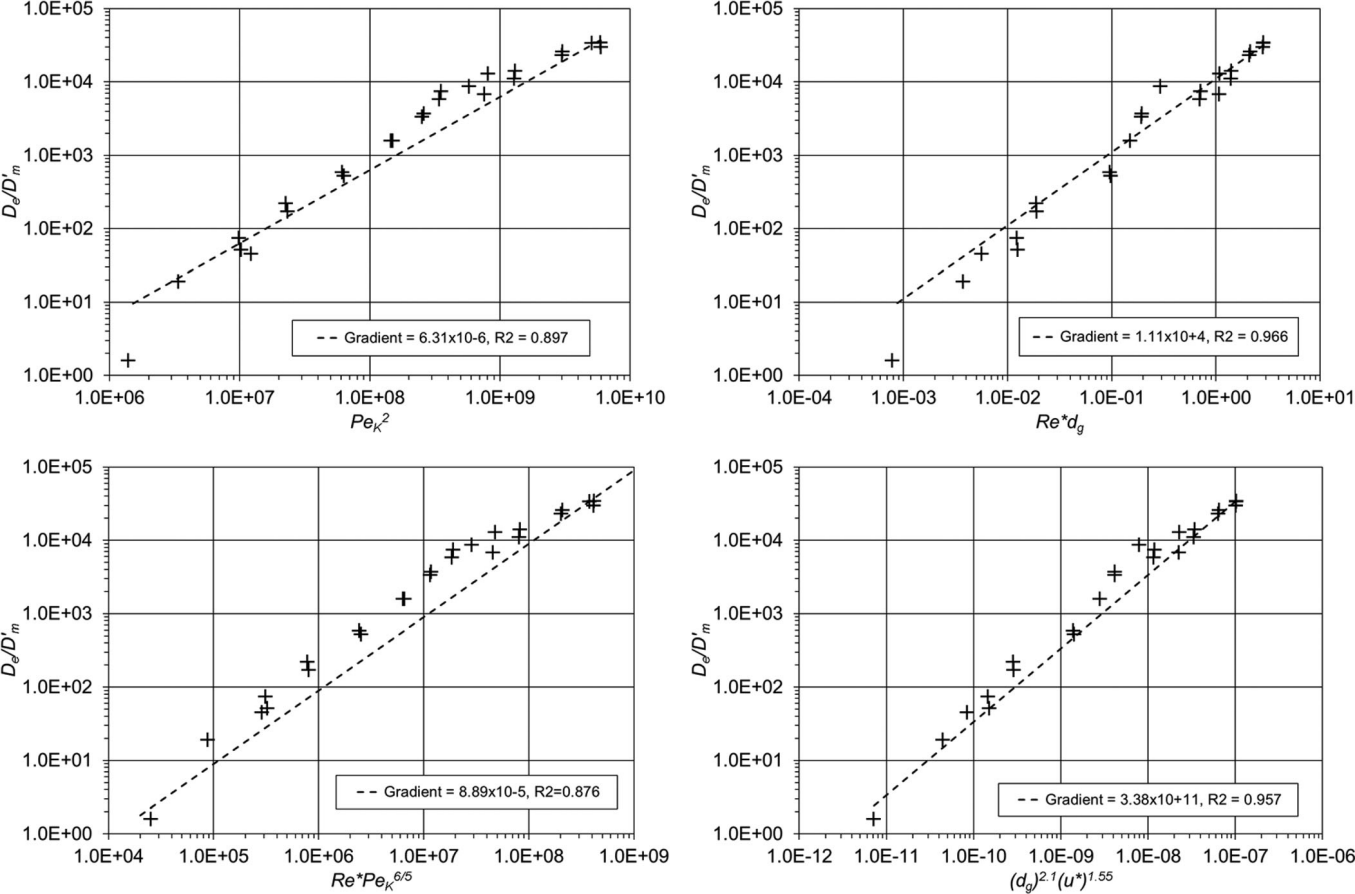
Comparison of water column derived exchange coefficients.

From flume data collected by *Richardson and Parr* [1988], a linear relationship was proposed to the square of the permeability Péclet number, arguing that the exchange processes were dominated by shear induced flow, and produced a gradient of 6.59 × 10^−5^. Assuming the same relationship, the new erosimeter data, which includes particles of greater diameter, gives a best fit linear relationship gradient of 6.31 × 10^−6^ (R^2^ = 0.897), shown in Figure 5a. As with the original data set, it is the extreme values which are limiting. Excluding the small diameter low shear stress and the large diameter high shear stress tests, produces a gradient of 1.12 × 10^−5^, though does not improve the goodness of fit (R^2^ = 0.874).

*Packman and Salehin* [2003] suggested that the interface mixing coefficient was proportional to the square of both the stream Reynolds number and the particle diameter. Investigating this relationship for the erosimeter data is not possible as there is no stream velocity or flow depth. Instead, replacing the stream Reynolds number with the shear Reynolds number, $Re_{*}$ and the flow depth with the roughness height allows a similar approach to be adopted. The best fit relationship using these parameters is linear, Figure 5b, having a gradient of 1.11x10^4^ (R^2^ = 0.966).

The repeatability of the erosimeter tests is better than previous experimental studies collated by *O'Connor and Harvey* [2008]. The new interface mixing coefficients lie within the scatter of the previous experimental data however they are consistently lower than the proposed scaling relationship of gradient of 5.0 × 10^−4^. This is most pronounced at the extremes, where combinations of either large diameter sediment and high bed shear velocity or small diameter and low bed shear velocities have been used. The coefficients, nondimensionalized using molecular diffusion, plotted against $Re_{*}Pe_{K}^{6/5}$ in Figure 4c, exhibit a trend similar to that shown by *O'Connor and Harvey* [2008]. The gradient of the best fit linear relationship between the parameters is 8.89 × 10^−5^ (R^2^ = 0.876).

Taking the new interface mixing coefficients obtained from the erosimeter experiments, from a range of particle diameters and bed shear velocities, with the permeability Péclet number, $Pe_{K}$ between 1000 and 77,000 and the shear Reynolds number, $Re_{*},$ between 5 and 600, a multiple linear regression analysis was performed to determine the best fit values assuming a relationship of the form $D_{e}/D_{m}^{\prime }={\alpha (d_{g})}^{\beta }{\left(u^{*}\right)}^{\gamma }$. The resulting relationship

(28) $$D_{e}/D_{m}^{\prime }={3.38\;x10^{11}\left(d_{g}\right)}^{2.1}{\left(u^{*}\right)}^{1.55}$$

with R^2^ = 0.957, is shown in Figure 5d. Expanding the relationship proposed by *O'Connor and Harvey* [2008] leads to values of 2.2 for both parameters *β* and *γ*, whereas the new relationship, derived directly from erosimeter data, suggests that the effect of bed shear velocity is less, with mean particle diameter having a greater influence. This is further supported by the relationship shown in Figure 5b, where values of 2.0 and 1.0 for the powers of *d_g_* and u* respectively provide a good fit to the data. Overall this suggests that for flat beds comprising uniform particle diameter, the interface mixing coefficient is affected to a greater extent by the particle diameter than bed shear velocity.

### 6.2 In‐Bed Studies

**Figure 6:**
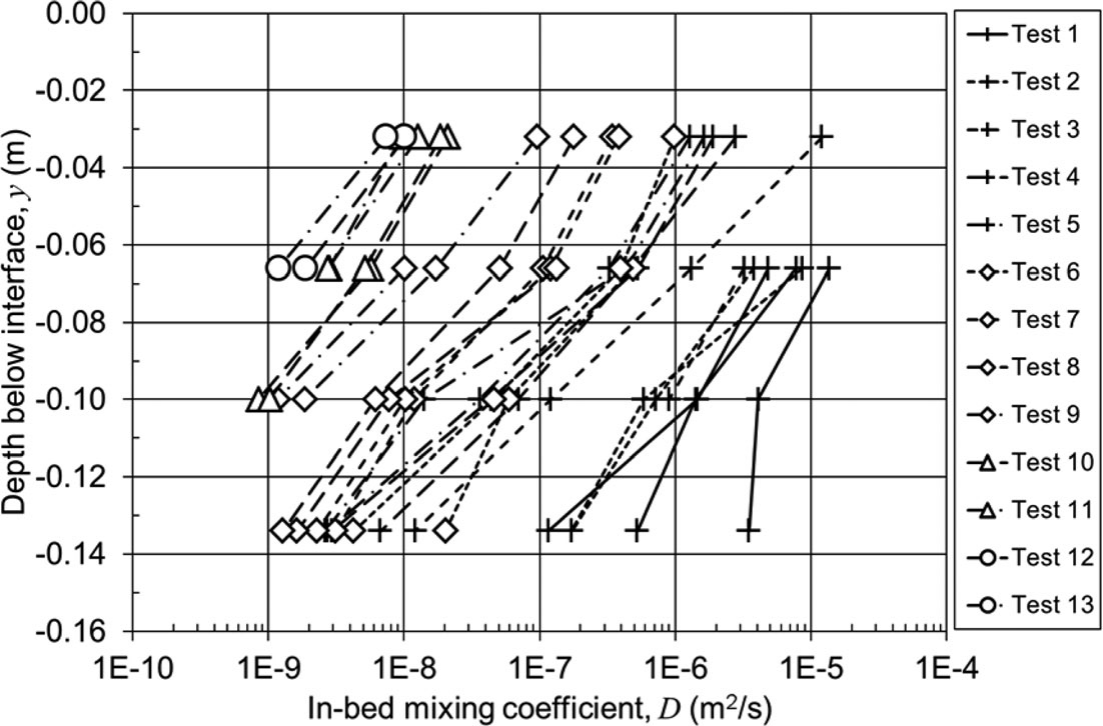
In‐bed mixing coefficients.

The in‐bed mixing coefficients are plotted in Figure 6 at the midpoint between the two profiles used to obtain the coefficient. There is good correlation between repeat tests and there is a clear variation of the mixing coefficient with depth below the interface. The effect of different mean sediment diameters and bed shear velocities is evident. There is more variation in the high permeability, high bed shear velocity experiments, on the right hand side of Figure 6, which is probably due to the higher coefficients and the slight variability in setup during these initial experiments. There is an almost constant exponential reduction of the mixing coefficient with depth, which appears to be independent of the experimental parameters of mean sediment diameter, $d_{g}$ and bed shear velocity, $u_{*}$, covering four orders of magnitude.

**Figure 7:**
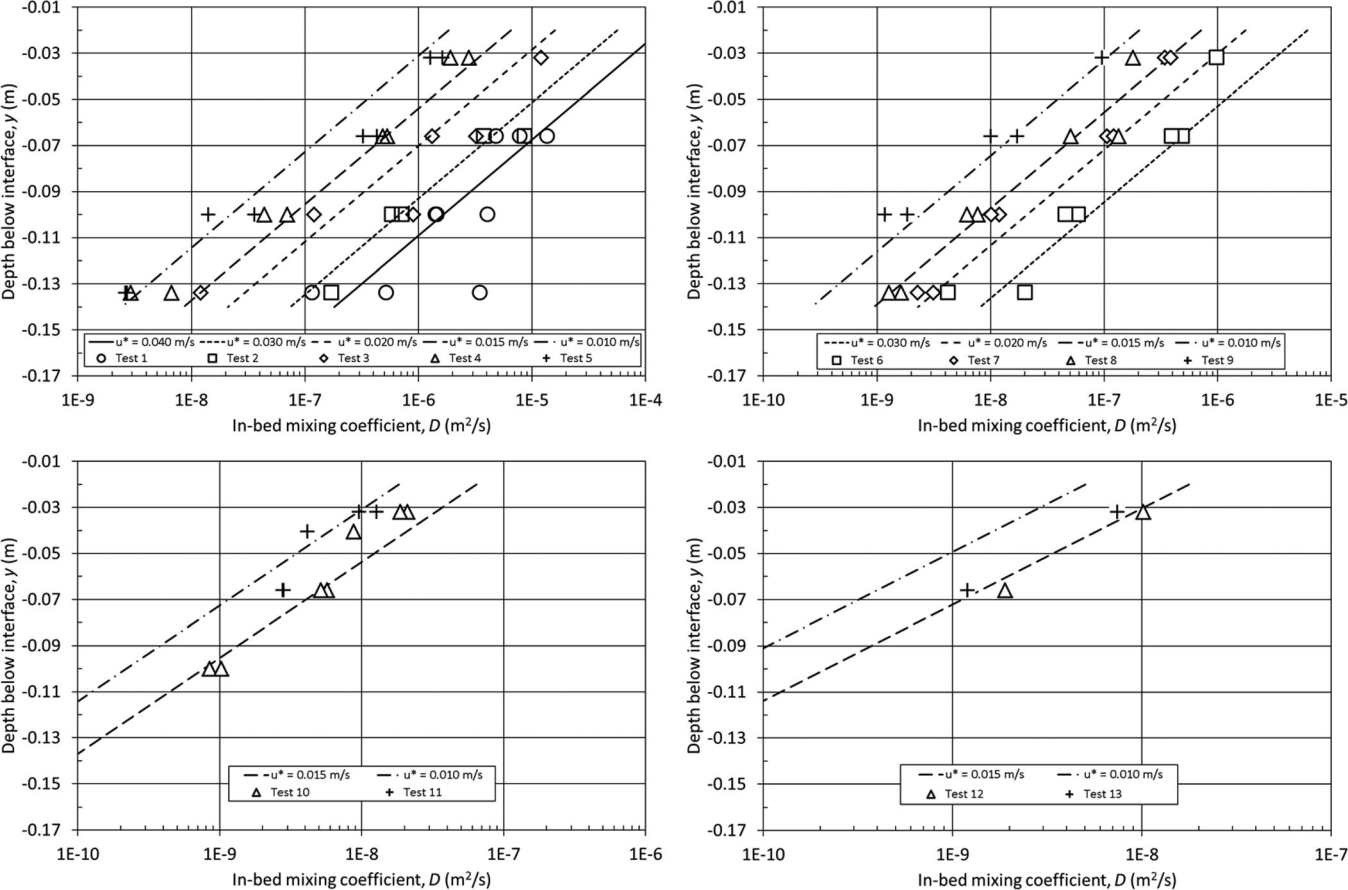
Predicted vertical variation of in‐bed mixing coefficients.

A multiple linear regression analysis was performed to determine the best fit values for the constants assuming a relationship to the experimental parameters of the form $D={\alpha (d_{g})}^{\beta }{\left(u^{*}\right)}^{\gamma }e^{\delta y}$. The resulting relationship is

(29) $$D={1.19x10^{6}(d_{g})}^{2.22}{\left(u^{*}\right)}^{3.11}e^{55y}$$

and is shown in Figure 7 for each of the mean particle diameters studied across the range of bed shear velocities, together with the evaluated in‐bed mixing coefficients. These show good agreement, with the majority of the data points falling within the range of bed shear velocities. Equation (29) can be used in conjunction with a 1‐D diffusion model to predict the temporal and spatial concentrations within the erosimeter. This assumes that the source of the mixing is from turbulence generated at the sediment‐water interface and that the turbulent fluctuations dissipate with distance below the interface. These processes are analogous to Fickian diffusion. As these tests have not been conducted for stratified, mixed grain or natural, angular sediments, there are limitations to the applicability of these results to natural aquatic systems. However, as a first approximation a relationship has been derived with the empirical permeability parameter, K, which produces the same vertical spatial variation and dependence on bed shear velocity, giving

(30) $$D={3.15x10^{11}(K)}^{1.32}{\left(u^{*}\right)}^{3.11}e^{55y}$$

Clearly if the permeability of the bed sediment varies spatially it will influence these estimations, reduced permeability likely reducing the depth of influence.

Comparing the relationships produced from multiple linear regressions for the interface exchange coefficient and the in‐bed mixing coefficient, (28) and (29), it is interesting to note that variation with mean particle diameter is very similar, the main difference being provided by the greater effect of the bed shear velocity on the spatial variation. Also there is between a half and one order of magnitude difference between the magnitude of the interface mixing coefficient and the in‐bed mixing coefficients closest to the interface. The interface mixing coefficient, calculated from the water column data, is a function of the exchange throughout the top portion of the bed and not therefore strictly the coefficient at the specific level of the interface. This is true of all the coefficients calculated using the initial slope method [*O'Connor and Harvey*, 2008], as a change in concentration is required to obtain the initial slope. This change in concentration means that a certain depth of interstitial fluid must mix with the water column. It is therefore unsurprising that the water column data give a lower coefficient than in‐bed measurements near the sediment‐water interface.

## 7 Conclusions

The aims of this study were to investigate the applicability of the erosimeter in studying hyporheic exchange and to obtain the vertical variation in mixing coefficient within flat sediment beds without sediment motion. A unique data set has been generated from which the vertical variation in mixing coefficient within a sediment bed exposed to turbulence driven hyporheic exchange has been evaluated.

The original EROSIMESS‐system (erosimeter) was redesigned to improve its use within a laboratory environment and to incorporate an in situ permeability test and fiber‐optic measurement system within the bed sediment. The flow field within the erosimeter was evaluated using particle image velocimetry (PIV), which validated the calibration between the propeller speed and bed shear velocity and demonstrated the uniformity of the flow field at the sediment water interface. Fiber‐optic fluorometers, developed for this study, have enabled concentration profiles to be measured within the bed sediment, permitting the vertical variation of in‐bed mixing coefficients with depth below the sediment‐water interface to be quantified.

Comparing the water column data derived interface mixing coefficients with different scaling relationships exhibits similar trends to previous studies, confirming that the erosimeter is a viable option for studying hyporheic exchange in the laboratory. Multiple linear regressions shows that the interface mixing coefficient is most accurately described by a function of the mean particle diameter, to the power 2.1 and the bed shear velocity to the power 1.55 within the range of the permeability Péclet number, $Pe_{K}$ between 1000 and 77,000 and the shear Reynolds number, $Re_{*},$ between 5 and 600.

The vertical variation of in‐bed mixing fits well to a constant exponential function over the full range of parameter combinations tested. A relationship to predict the spatial variation within a sediment bed has been developed and relies on the bed shear velocity and either the particle diameter or the permeability. Quantifying the variation in mixing coefficient below the sediment‐water interface will allow chemical concentrations within sediment beds to be modeled more accurately. The relationship enables predictions of the depth to which concentrations of pollutants will penetrate into the bed sediment, allowing the active layer (the region where exchange will occur faster than molecular diffusion) to be obtained. This is an important aspect for consideration in determining the ecological impact of in the exchange of dissolved oxygen, nutrient and anthropogenic inputs.

## Notation

$A_{s}$: Surface area of sediment bed, L^2^.
b,c: Equation constant
C: Solute concentration, ML^−3^.
$C_{0,s}$: Initial solute concentration within sediment pore water, ML^−3^.
D: Mixing coefficient, L^2^T^−1^.
$D_{e}$: Effective interface mixing coefficient, L^2^T^−1^.
$D_{m}^{\prime }$: Molecular diffusion coefficient in sediment pore water, L^2^T^−1^.
$D_{1}$: Average mixing coefficient of region between sensors, L^2^T^−1^.
$D_{2}$: Average mixing coefficient of region below sensors, L^2^T^−1^.
$d_{g}$: Geometric mean particle diameter, L.
$d_{10}$: Particle size for which 90% of sediment is coarser, L.
$d_{90}$: Particle size for which 90% of sediment is finer, L.
e: Base of natural logarithm.
g: Acceleration due to gravity, MLT^−2^.
h: flow depth, L.
I: Hydraulic gradient.
K: Permeability, L^2^.
$K_{c}$: Hydraulic conductivity, LT^−1^.
$k_{s}$: Roughness height, L.
L: Distance between sensors, L.
$M_{w}$: Mass accumulation in water, ML^−2^.
N: Number of vector fields.
$Pe_{K}$: Permeability Péclet number.
Q: Discharge, L^3^T^−1^.
$R_{T}$: Temperature correction factor.
$R_{t}^{2}$: Coefficient of determination.
Re: Stream Reynolds number.
$Re_{*}$: Shear Reynolds number.
t: time, T.
*U*: average main stream velocity, LT^−1^.
u′: Instantaneous velocity fluctuation in x‐direction, LT^−1^.
$\bar{u}$: Ensemble average velocity in x‐direction, LT^−1^.
$u_{*}$: Bed shear velocity, LT^−1^.
v′: Instantaneous velocity fluctuation in y‐direction, LT^−1^.
w′: Instantaneous velocity fluctuation in z‐direction, LT^−1^.
x: Horizontal coordinate, L.
y: Vertical coordinate or distance between manometer gland points, L.
z: Lateral horizontal coordinate, L.
Δ: Bed‐form height, L.
α, β, γ, δ: Equation constants.
ε: Dummy variable.
θ: Porosity.
λ: Bed‐form wavelength, L.
μ: Dynamic viscosity, ML^−1^T^−1^.
ν: Kinematic viscosity, L^2^T^−1^.
$\rho_{w}$: Fluid density, ML^−3^.
τ: Stress or bed shear stress, ML^−1^T^−2^.

## References

[wrcr22044-bib-0001] Bear, J. (1972), Dynamics of Fluids in Porous Media, Am. Elsevier, N. Y.

[wrcr22044-bib-0002] Boano, F. , J. W. Harvey , A. Marion , A. I. Packman , R. Revelli , L. Ridolfi , and A. Wörman (2014), Hyporheic flow and transport processes: Mechanisms, models, and biogeochemical implications, Rev. Geophys., 52, 603–679, doi:10.1002/2012RG000417.

[wrcr22044-bib-0003] Bottacin‐Busolin, A. , G. Singer , M. Zaramella , T. J. Battin , and A. Marion (2009), Effects of streambed morphology and biofilm growth on the transient storage of solutes, Environ. Sci. Technol., 43, 7337–7342. 19848143 10.1021/es900852w

[wrcr22044-bib-0004] British Standard (1990), BS1377‐5: Soils for Civil Engineering Purposes: Compressibility, Permeability and Durability Tests, Br. Standards Inst, London, U. K.

[wrcr22044-bib-0005] Carman, P. C. (1937), Fluid flow through granular beds, Trans. Inst. Chem. Eng., 15, 32–48.

[wrcr22044-bib-0006] Chandler, I. D. (2012), Vertical variation in diffusion coefficient within sediments, PhD thesis, Univ. of Warwick, Coventry, U. K.

[wrcr22044-bib-0007] Chandler, I. D. , J. M. Pearson , I. Guymer and R. van Egmond (2010), Quantifying hyporheic exchange coefficients using the EROSIMESS‐system, in 6th International Symposium on Environmental Hydraulics (ISEH), 25–26 June 2010, vol. 2, pp. 765–770, Taylor and Francis, Athens, Greece.

[wrcr22044-bib-0008] Cho, Y. M. , D. Werner , K. B. Moffett , and R. G. Luthy (2010), Assessment of advective porewater movement affecting mass transfer of hydrophobic organic contaminants in marine intertidal sediment, Environ. Sci. Technol., 44, 5842–5848. 20608739 10.1021/es903583y

[wrcr22044-bib-0009] Crank, J. , and P. Nicolson (1947), A practical method for numerical evaluation of solutions of partial differential equations of the heat‐conduction type, Proc. Cambridge Philos. Soc., 43, 50–67.

[wrcr22044-bib-0010] Elliott, A. H. , and N. H. Brooks (1997), Transfer of nonsorbing solutes to a streambed with bedforms: Theory, Water Resour. Res., 33, 123–136.

[wrcr22044-bib-0011] Fischer, H. B. , E. J. List , R. C. Y. Koh , J. Imberger and N. H. Brooks (1979), Mixing in Inland and Coastal Waters, Academic Press Inc., London, U. K.

[wrcr22044-bib-0012] Freeze, R. A. , and J. A. Cherry (1979), Groundwater, Prentice Hall, Inc, Englewood Cliffs, N. J.

[wrcr22044-bib-0013] Fries, J. S. (2007), Predicting interfacial diffusion coefficients for fluxes across the sediment‐water interface, J. Hydraul. Eng., 133, 267–272.

[wrcr22044-bib-0014] Habel, F. , C. Mendoza and A. C. Bagtzoglou (2002), Solute transport in open channel flows and porous streambeds, Adv. Water Resour., 25, 455–469.

[wrcr22044-bib-0015] Hart, D. R. (1995), Parameter estimation and stochastic interpretation of the transient storage model for solute transport in streams, Water Resour. Res., 31, 323–328.

[wrcr22044-bib-0016] Hester, E. T. , K. I. Young , and M. A. Widdowson (2013), Mixing of surface and groundwater induced by riverbed dunes: Implications for hyporheic zone definitions and pollutant reactions, Water Resour. Res., 49, 5221–5237, doi:10.1002/wrcr.20399.

[wrcr22044-bib-0017] Johansson, H. , K. Jonsson , K. J. Forsman , and A. Wörman (2001), Retention of conservative and sorptive solutes in streams ‐ simultaneous tracer experiments, Sci. Total Environ., 266, 229–238. 11258821 10.1016/s0048-9697(00)00758-0

[wrcr22044-bib-0018] Jonsson, K. , H. Johansson , and A. Wörman (2003), Hyporheic exchange of reactive and conservative solutes in streams ‐ tracer methodology and model interpretation, J. Hydrol., 278, 153–171.

[wrcr22044-bib-0019] Jubb, S. , I. Guymer , G. Licht , and J. Prochnow (2001), Relating oxygen demand to flow: Development of an in‐situ sediment oxygen demand measurement device, Water Sci. Technol., 43, 203–210. 11379133

[wrcr22044-bib-0020] Liem, R. , V. Spork , and J. Koengeter (1997), Investigations on erosional processes of cohesive sediment using an in‐situ measuring device, Int. J. Sediment Res., 13, 139–147.

[wrcr22044-bib-0021] Liu, H. H. , L. J. Bao , K. Zhang , S. P. Xiu , F. G. Wu , and E. Y. Zeng (2013), Novel passive sampling device for measuring sediment‐water diffusion fluxes of hydrophobic organic chemicals, Environ. Sci. Technol., 47, 9866–9873. 23919591 10.1021/es401180y

[wrcr22044-bib-0022] Liu, H. H. , L. J. Bao , and E. Y. Zeng (2014), Recent advances in the field measurement of the diffusive flux of hydrophobic organic chemicals at the sediment‐water interface, Trends Anal. Chem., 54, 56–64.

[wrcr22044-bib-0023] Marion, A. , M. Bellinello , I. Guymer , and A. I. Packman (2002), Effect of bed form geometry on penetration of nonreactive solute into a streambed, Water Resour. Res., 38(10), 1209, doi:10.1029/2001WR000264.

[wrcr22044-bib-0024] Marion, A. , M. Zaramella , and A. I. Packman (2003), Parameter estimation of the transient storage model for stream‐subsurface exchange, J. Environ. Eng., 129, 456–463.

[wrcr22044-bib-0025] McAvoy, D. C. , P. Masscheleyn , C. Peng , S. W. Morrall , A. B. Casilla , J. M. U. Lim , and E. G. Gregorio (2003), Risk assessment approach for untreated wastewater using the QUAL2E water quality model, Chemosphere, 52, 55–66. 12729687 10.1016/S0045-6535(03)00270-4

[wrcr22044-bib-0026] Nagaoka, H. , and S. Ohgaki (1990), Mass transfer mechanism in a porous riverbed, Water Res., 24, 417–425.

[wrcr22044-bib-0027] O'Connor, B. L. , and J. W. Harvey (2008), Scaling hyporheic exchange and its influence on biogeochemical reactions in aquatic ecosystems, Water Resour. Res., 44, W12423, doi:10.1029/2008WR007160.

[wrcr22044-bib-0029] Packman, A. I. , and M. Salehin (2003), Relative roles of stream flow and sedimentary conditions in controlling hyporheic exchange, Hydrobiologia, 494, 291–297.

[wrcr22044-bib-0030] Packman, A. I. , M. Salehin and M. Zaramella (2004), Hyporheic exchange with gravel beds: Basic hydrodynamic interactions and induced advective flows, J. Hydraul. Eng., 130, 647–656.

[wrcr22044-bib-0031] Rehg, K. J. , A. I. Packman , and J. Ren (2005), Effects of suspended sediment characteristics and bed sediment transport on streambed clogging, Hydrol. Processes, 19, 413–427.

[wrcr22044-bib-0032] Ren, J. , and A. I. Packman (2004), Stream‐subsurface exchange of zinc in the presence of silica and kaolinite colloids, Environ. Sci. Technol., 38, 6571–6581. 15669314 10.1021/es035090x

[wrcr22044-bib-0033] Richardson, C. P. , and A. D. Parr (1988), Modified Fickian model for solute uptake by runoff, J. Environ. Eng., 114, 792–809.

[wrcr22044-bib-0034] Runkel, R. L. (1998), One dimensional transport with inflow and outflow (OTIS): A solute transport model for streams and rivers, *U.S. Geol. Surv. Water Resour. Invest. Rep*., 98‐4018, 73 pp.

[wrcr22044-bib-0035] Shimizu, Y. , T. Tsujimoto and H. Nakagawa (1990), Experiment and macroscopic modelling of flow in highly permeable porous medium under free‐surface flow, Hydrosci. Hydraul. Eng., 8, 69–78.

[wrcr22044-bib-0036] Smart, P. L. , and I. M. S. Laidlaw (1977), An evaluation of some fluorescent dyes for water tracing, Water Resour. Res., 13, 161–172.

[wrcr22044-bib-0037] Spork, V. , J. Jahnke , J. Prochnow , and J. Koengeter (1997), Stabilising effect of benthic algae on cohesive sediments, Int. J. Sediment Res., 12, 399–406.

[wrcr22044-bib-0038] Technical Guidance Document (TGD) (2003), *Technical Guidance Document in Support of Commission Directive 93/67/EEC on Risk Assessment for New Substances and Commission Regulation (EC) No* *1488/94 on Risk Assessment for Existing Substances*, European Commission Joint Research Centre.

[wrcr22044-bib-0039] Tennekes, H. , and J. L. Lumley (1972), A First Course in Turbulence, MIT Press, Boston, USA.

[wrcr22044-bib-0040] Tonina, D. , and J. M. Buffington (2007), Hyporheic exchange in gravel bed rivers with pool‐riffle morphology: Laboratory experiments and three‐dimensional modelling, Water Resour. Res., 43, W01421, doi:10.1029/2005WR004328.

[wrcr22044-bib-0041] van Rijn, L. C. (1984), Sediment transport, part III: Bed forms and alluvial roughness, J. Hydraul. Eng., 110, 1733–1754.

[wrcr22044-bib-0042] Wilson, A. M. , M. Huettel , and S. Klein (2008), Grain size and depositional environment as predictors of permeability in coastal marine sands, Estuarine Coastal Shelf Sci., 80, 193–199.

[wrcr22044-bib-0043] Wörman, A. (2000), Comparison of models for transient storage of solutes in small streams, Water Resour. Res., 36, 455–468.

[wrcr22044-bib-0044] Wörman, A. , A. I. Packman , H. Johansson , and K. Jonsson (2002), Effect of flow‐induced exchange in hyporheic zones on longitudinal transport of solutes in streams and rivers, Water Resour. Res., 38(1), 1001, doi:10.1029/2001WR000769.

[wrcr22044-bib-0045] Young, P. , A. Jakeman , and R. McMurtrie (1980), An instrumental variable method for model order identification, Automatica, 16, 281–294.

